# Genome-Wide Identification of Sorghum Paclobutrazol-Resistance Gene Family and Functional Characterization of *SbPRE4* in Response to Aphid Stress

**DOI:** 10.3390/ijms25137257

**Published:** 2024-07-01

**Authors:** Yongchao Guo, Zhifang Wang, Zhiyin Jiao, Guang Yuan, Li Cui, Pengwei Duan, Jingtian Niu, Peng Lv, Jinping Wang, Yannan Shi

**Affiliations:** 1 Institute of Millet Crops, Hebei Academy of Agriculture & Forestry Sciences/Hebei Branch of China National Sorghum Improvement Center, Shijiazhuang 050035, China; gycxhxbc115@163.com (Y.G.); cctvwzf@126.com (Z.W.); zhiyin_jiao@163.com (Z.J.); yuanguang@haafs.org (G.Y.); 108545810@126.com (J.N.); pengl001@126.com (P.L.); 2 Hebei Plant Protection and Plant Inspection Station, Shijiazhuang 050035, China; gycxhxbc@163.com; 3 Hebei Academy of Agriculture & Forestry Sciences, Shijiazhuang 050035, China; nkydpw@126.com

**Keywords:** *Paclobutrazol-Resistance* (*PRE*), sorghum (*Sorghum bicolor*), genome-wide identification, aphid stress

## Abstract

Sorghum (*Sorghum bicolor*), the fifth most important cereal crop globally, serves as a staple food, animal feed, and a bioenergy source. *Paclobutrazol-Resistance* (*PRE*) genes play a pivotal role in the response to environmental stress, yet the understanding of their involvement in pest resistance remains limited. In the present study, a total of seven *SbPRE* genes were found within the sorghum BTx623 genome. Subsequently, their genomic location was studied, and they were distributed on four chromosomes. An analysis of cis-acting elements in *SbPRE* promoters revealed that various elements were associated with hormones and stress responses. Expression pattern analysis showed differentially tissue-specific expression profiles among *SbPRE* genes. The expression of some *SbPRE* genes can be induced by abiotic stress and aphid treatments. Furthermore, through phytohormones and transgenic analyses, we demonstrated that *SbPRE4* improves sorghum resistance to aphids by accumulating jasmonic acids (JAs) in transgenic Arabidopsis, giving insights into the molecular and biological function of atypical basic helix-loop-helix (bHLH) transcription factors in sorghum pest resistance.

## 1. Introduction

Sorghum (*Sorghum bicolor*) is the fifth cereal crop in terms of total production worldwide, providing a staple food source for over half a billion people in Africa and Asia, a nutritional supplement for livestock, and a biofuel resource for industrial use [1]. It also contributes to the production of industrial materials such as alcohol [2]. Previous studies indicate that sorghum stems can contain approximately 20% sugar under stress conditions, a component directly convertible to biofuel [2]. The resilience of various sorghum germplasms to both biotic and abiotic stresses further enhance its value [3]. Consequently, sorghum presents as an optimal energy crop for cultivation on nutrient-deficient soils, promising to improve the productivity of agriculture. However, its stable production is seriously threatened by the sorghum aphid (*Melanaphis sacchari*) [4]. The aphid can feed on the entire reproductive period of sorghum. They tend to directly cluster and parasitize on the stems and underside of leaves, and in severe cases, they can cover the entire sorghum plant. While sucking the sap from the sieve tubes, sorghum aphids also secrete ‘honeydew’, which covers the leaves, reduces plant photosynthesis, and affects metabolic reactions. Affected plants will show a loss of green color in leaves, and stems become fragile, making them more prone to lodging. Additionally, the sorghum aphid can also transmit viruses such as maize red stripe, sugarcane mosaic, and sugarcane yellow leaf viruses, leading to reduced sorghum yield and lower quality [5].

The bHLH is one of the most important transcription factor (TF) families in plants [6], containing a basic DNA-binding domain and a highly conserved helix–loop–helix domain [7,8]. Based on their DNA-binding capability, bHLH proteins can be divided into DNA-binding bHLH (typical bHLH) and non-DNA-binding bHLH (atypical HLH) [8]. Atypical bHLH proteins lack the basic region necessary for DNA binding, yet their HLH domain can engage with typical bHLH proteins to modulate downstream genes [9,10]. 

The PRE proteins are classified as atypical bHLH transcription factors and have been implicated in the regulation of plant growth and development. Additionally, they have been shown to participate in responses to plant hormones and environmental stimuli, such as temperature and light [9,10,11,12,13,14]. *Arabidopsis thaliana* has six *PRE* genes that display a range of functions in the development of plants. PRE1/BANQUO1/(BNQ1)/bHLH136, firstly identified as PRE transcription factor, influenced processes such as seed germination, stem/leaf elongation, flowering, and fruit development through GA signaling [9,14,15,16]. Similarly, other PRE proteins such as PRE3, PRE4, and PRE6 play roles in various physiological processes, including plant hormones signaling, light response, and organ elongation [13,17]. Furthermore, the regulation of *PRE* genes by transcription factors such as BRASSINAZOLE-RESISTANT1 (BZR1) and phytochrome-interacting factor 4 (PIF4) underscores their involvement in multiple signaling pathways and developmental processes in plants [11,16].

Previous research has focused on the function of *PRE* genes in plant growth, development, and abiotic stress tolerance. To elucidate the roles of *PRE* genes in sorghum aphid stress response, seven PRE members in sorghum were identified by homologous sequence alignment. Additionally, this study characterized the *SbPRE* gene family using bioinformatics and molecular biology techniques, including an analysis of genetic structure, promoter regions, and expression patterns. The results indicated that *SbPRE* genes, especially *SbPRE4*, are involved in the aphid stress response, and this study establishes the foundation for further investigations into the biological and molecular functions of *SbPRE* genes.

## 2. Results

### 2.1. Identification and Characterization of the PRE Gene Family in the Sorghum Genome

**Figure 1:**
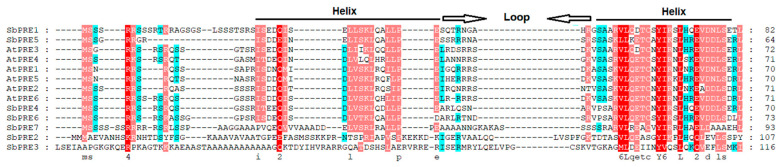
The amino acid sequence alignment of the SbPRE and AtPRE proteins. The helix–loop–helix conserved motif is indicated. The colors background indicated identical amino acids.

According to Fan et al. [18], the sorghum genome contains 174 *bHLH* genes. To identify sorghum PRE members in this study, BLASTp analysis was performed utilizing *Arabidopsis thaliana* PRE protein sequences. Through this analysis, seven SbPRE members were screened out in the BTx623 genome and assigned the names *SbPRE1*, *SbPRE2*, *SbPRE3*, *SbPRE4*, *SbPRE5*, *SbPRE6*, and *SbPRE7* according to their sequence similarity with *A. thaliana PRE* genes. (Table 1). The CDS lengths and protein lengths of the seven identified *SbPRE* genes varied from 261 to 1080 bp and 87 to 360 amino acids, respectively (Table 1). The N-terminal regions of all SbPRE proteins were found to contain the highly conserved bHLH domain (Table 1, Figure 1). The molecular weight of the SbPRE proteins were predicted to range from 9690.97 (SbPRE5) to 37,275.27 (SbPRE3) Da. Furthermore, the predicted isoelectric points (pI values) of these proteins varied between 6.35 (SbPRE3) and 9.48 (SbPRE2) based on sequence analysis.

### 2.2. Chromosome Localization, Gene Structure, and Genetic Evolution Analysis of the SbPRE Genes

**Figure 2:**
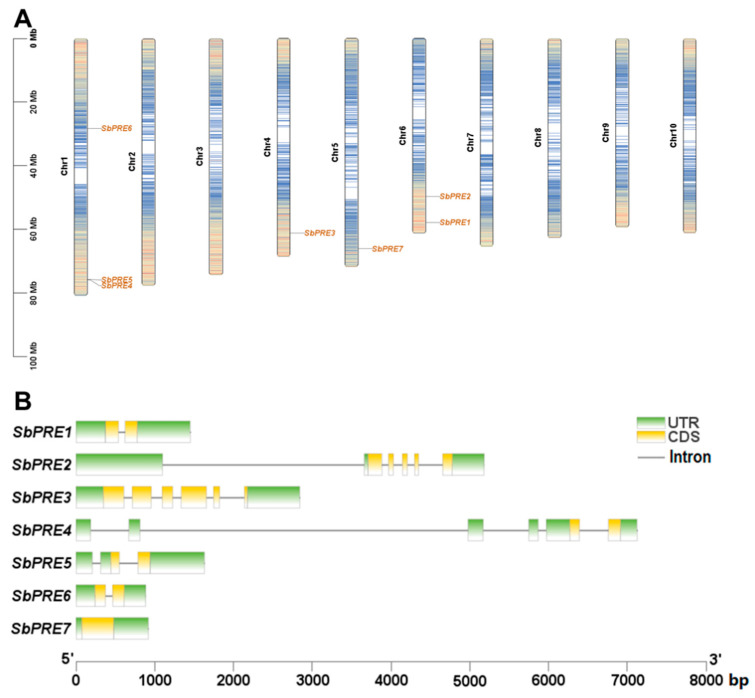
The location of *SbPRE* genes on chromosomes and gene structures (introns/exons). (**A**) Chromosomal location of seven *SbPREs* on chromosomes. Colors represent gene density, with blue indicating low density and yellow indicating high density. (**B**) Gene structures (introns/exons) of the *SbPRE* genes.

Genomic location data, retrieved from the Phytozome v13 database, facilitated the mapping of *SbPRE* genes onto four distinct chromosomes. Chromosome 4 and chromosome 5 individually harbor one *SbPRE* gene, while chromosome 6 hosts *SbPRE1* and *SbPRE2*, and chromosome 1 contains *SbPRE4*, *SbPRE5*, and *SbPRE6* (Figure 2A). Details regarding the IDs and genomic coordinates of these *SbPRE* genes are presented in Table 1.

To identify the distinctions among *SbPRE* gene architectures, the numbers of exons and introns were analyzed. The results showed that *SbPRE1*, *SbPRE4*, *SbPRE5*, and *SbPRE6* contain two exons and one intron, which were similar to the architecture of *MdPRE* genes [19], while *SbPRE2*, *SbPRE3*, and *SbPRE7* contain five, six, and one exon(s), respectively, (Figure 2B), indicating the functional differentiation of the *SbPRE* gene during evolution.

**Figure 3:**
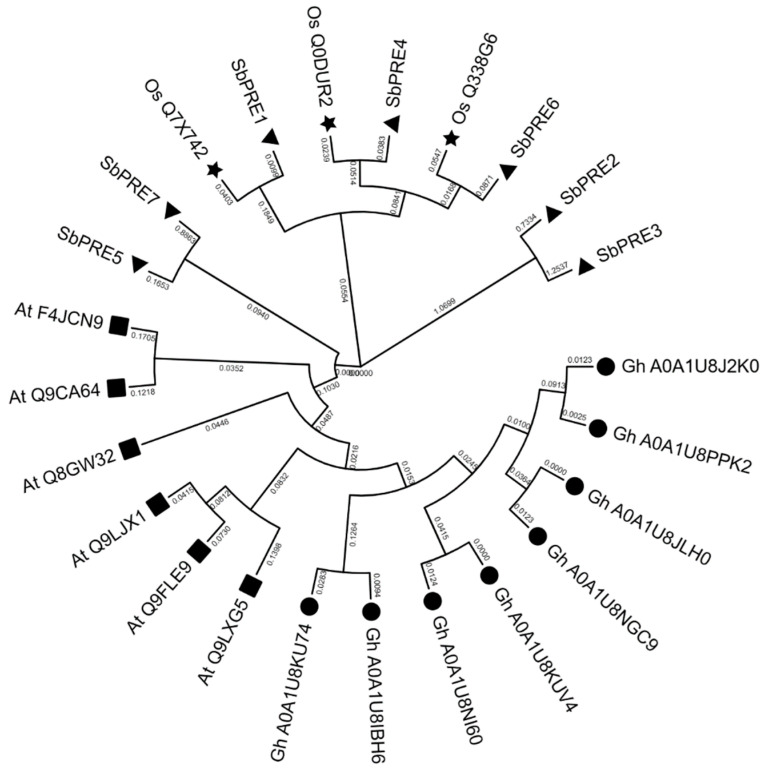
Phylogenetic analysis of PRE proteins in sorghum, Arabidopsis, rice, and cotton. Triangle represents SbPRE protein, star represents OsPRE protein, rect represents AtPRE protein, and circle represents GhPRE protein.

Phylogenetic analysis is crucial for elucidating the evolutionary relationships among crop species. A phylogenetic tree was generated through the utilization of seven PRE proteins from sorghum (*Sorghum bicolor*), five PRE proteins from Arabidopsis (*Arabidopsis thaliana*), and three PRE proteins from rice (*Oryza sativa*) in conjunction with eight PRE proteins in cotton (*Gossypium hirsutum*). The resultant phylogenetic tree showed that SbPRE1 was closely related to Os_Q7X742, SbPRE4 was more closely related to Os_Q0DUR2, and SbPRE6 was closely related to Os_Q338G6 (Figure 3), which suggested SbPREs might have a similar biological function to their homologs. However, SbPRE2, SbPRE3, and SbPRE5, SbPRE7, were clustered into independent groups, respectively, (Figure 3), indicating that they might modulate the important agronomy traits related to sorghum evolution and domestication.

### 2.3. Expression Patterns of SbPREs across Sorghum Tissues

**Figure 4:**
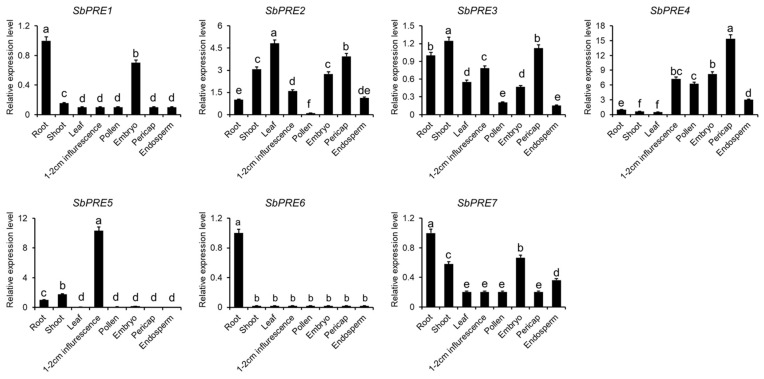
The relative expression analysis of *PRE* genes in sorghum tissues. The RNA-seq data of different sorghum tissues were obtained from Wang et al. [20]; the study accession in EBI database (https://www.ebi.ac.uk/ena/browser/view/ (accessed on 21 May 2024)) is ERP024458. Relative expression of the *SbPREs* was measured in sorghum root, shoot, leaf, seedling, 1–2 cm inflorescence, pollen, embryo, endosperm, and pericarp. Different lowercase letters indicate a significant difference at *p* < 0.05 based on one-way analysis of variance (ANOVA).

For the initial exploration of temporal and spatial expression profiles of *SbPRE* genes across sorghum tissues, the levels of *SbPRE* expression were evaluated in root, shoot, leaf, seedling, 1–2 cm inflorescence, pollen, embryo, endosperm, and pericarp using public data (Figure 4) [20]. The results showed that the *SbPRE* genes were expressed in most tissues. *SbPRE2*, *SbPRE3*, and *SbPRE7* exhibited high expression levels in root, shoot, leaf, inflorescence, and seed organs, suggesting their significant roles during growth and developmental processes. *SbPRE1* and *SbPRE6* demonstrated peak expression in root. Interestingly, *SbPRE4* and *SbPRE5* displayed a substantial up-regulation in inflorescence. Particularly, *SbPRE4* also showed predominant expression in pollen and seed organs, indicating their likely involvement in the reproductive growth stage. 

### 2.4. Analysis of Cis-Elements in SbPREs Promoter

**Figure 5:**
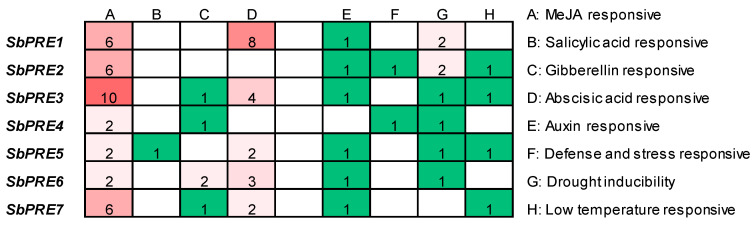
The *cis*-elements analysis of *SbPRE* promoters. 2 kb sequences upstream of the start codon were used for analysis.

The expression and function of genes are influenced by cis-regulatory elements present in their promoter regions. In this study, cis-acting elements within the 2 kb promoter sequences upstream of *SbPRE* genes were examined to identify elements associated with environmental stresses and hormone responses using the PlantCARE database [21] (Figure 5A). A total of 81 cis-elements were predicted and categorized into eight distinct types: (1) auxin-responsive elements such as the TGA-element and AuxRR-core; (2) defense and stress response-related TC-rich repeats; (3) elements linked to gibberellin (GA) responses including the TATC-box, P-box, and GARE-motif; (4) low-temperature responsive LTR elements; (5) salicylic acid (SA)-responsive TCA-element; (6) ABA-responsive ABRE transcriptional factor; (7) anaerobic and anoxic response-inducing ARE and GC-motif elements; and (8) methyl-jasmonic acid (MeJA) response-associated CGTCA- and TGACG-motifs. Notably, each *SbPRE* gene promoter harbored a minimum of two types of these cis-elements, with the MeJA responsive element being the most prevalent element identified. All of the above suggested that *SbPRE* genes might play vital roles in sorghum adaption to environmental challenges.

### 2.5. SbPRE Genes Expression under Abotic and Aphid Stress

**Figure 6:**
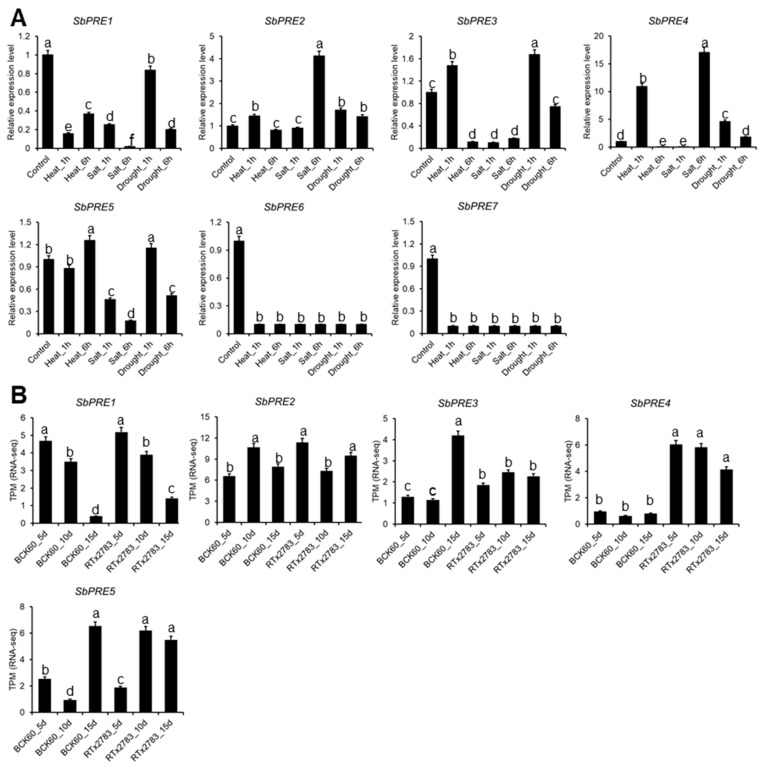
*SbPRE* genes expression analysis under different stresses by public RNA-seq data [22,23]. (**A**) The relative expression levels under heat, salt, and drought stress. (**B**) Expression levels (TPM) of *SbPRE* genes responsive to aphid stress. Data are mean ± SD (n = 3). Different lowercase letters indicate a significant difference at *p* < 0.05 based on one-way analysis of variance (ANOVA).

In order to identify the roles of *SbPREs* in response to abiotic stress (heat, salt, and drought) and biotic stress (aphid), RNA-Seq data under heat, salt, drought, and aphid stress treatment [22], ref. [23] were reanalyzed using Tbtools. Notably, under three abiotic stress conditions, *SbPRE1*, *SbPRE6*, and *SbPRE7* exhibited down-regulation, whereas *SbPRE2* and *SbPRE4* demonstrated up-regulation at different time points. *SbPRE3* and *SbPRE5* showed induced expression in response to heat and drought stress (Figure 6A). Under aphid stress, *SbPRE1* was down-regulated at the 10th and 15th day after aphid inoculation in both BCK60 (aphid-susceptible material) and RTx2783 (aphid-resistant material). *SbPRE2* showed the opposite expression pattern in susceptible (BCK60) and resistant (RTx2783) lines. After 15-day aphid inoculation, *SbPRE3* and *SbPRE3* exhibited obvious up-regulation in BCK60. It is notable that under aphid treatment, *SbPRE4* exhibited higher expression in the resistant line of RTx2783 compared to BCK60 (Figure 6B). The induced expression patterns suggest a potential role for *SbPREs* in stress adaptation mechanisms in sorghum.

### 2.6. Overexpressing SbPRE4 Enhanced Aphid Resistance of Arabidopsis Thaliana by Accumulating JAs

**Figure 7:**
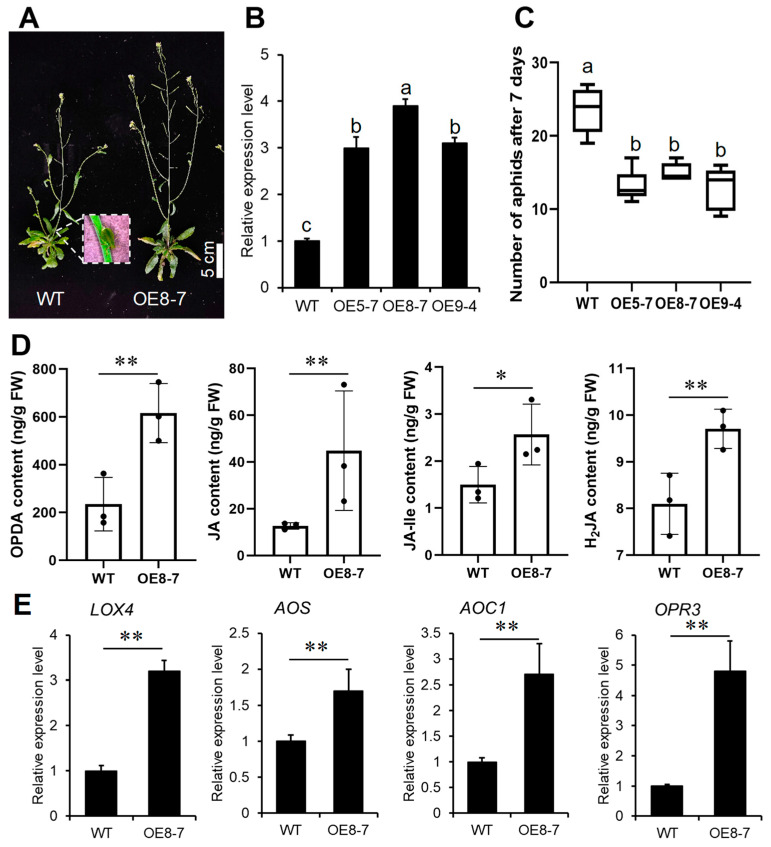
*SbPRE4* improves aphid resistance of Arabidopsis by JAs. (**A**) Phenotypic comparison of 28-day-old Col-0 and OE *SbPRE4* transgenic Arabidopsis after 7-day aphid infestation. (**B**) Expression analysis of *SbPRE4* in Col-0 and OE *SbPRE4* transgenic Arabidopsis. (**C**) Number of aphids on Col-0 and OE *SbPRE4* transgenic Arabidopsis after 7-day infestation. (**D**) JA, OPDA, JA-Ile, and H_2_JA content in 28-day-old Col-0 and OE *SbPRE4* transgenic Arabidopsis. (**E**) Expression analysis of JA biosynthesis-related genes in Col-0 and OE *SbPRE4* transgenic Arabidopsis. (**B**,**C**) values represent means ± SD, n = 3 plants. Different lowercase letters indicate a significant difference at *p* < 0.05 based on one-way analysis of variance (ANOVA). Student’s *t*-test (* *p* < 0.05 and ** *p* < 0.01) was used to analyze statistical significance of (**D**,**E**).

In order to assess the potential aphid resistance conferred by the heterologous expression of *SbPRE4* in Arabidopsis, we generated *SbPRE4* over-expression (OE) stable transgenic lines by transforming *p35S::SbPRE4 into Arabidopsis thaliana* (Col-0). Homozygous plants from three independent transformed lines (OE 5-7, OE 8-7, and OE 9-4) were obtained, and the expression of *SbPRE4* was validated using qRT-PCR (Figure 7B). An evaluation of *M. persicae* proliferation revealed a significant decrease in aphid densities in these three transgenic lines compared to Col-0 (Figure 7A,C). These findings provide additional evidence supporting the proposition that improved *SbPRE4* expression can effectively reduce the aphid population.

**Figure 8:**
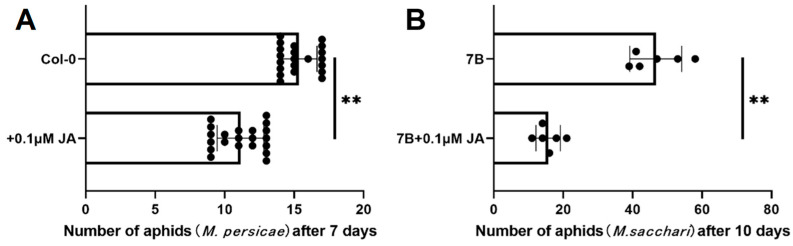
Number of aphids on JA-treated Col-0 (**A**) and 7B (**B**) after aphid infestation. Values represent means ± SD, n = 21 (Arabidopsis) and 6 (sorghum) plants. Student’s *t*-test: ** *p* < 0.01.

Jasmonic acids contribute to plant tolerance to aphids [24]. We measured the content of JA, OPDA, JA-Ile, and H_2_JA in Col-0 and the transgenic lines. The results demonstrate that *SbPRE4* transgenic lines exhibited elevated levels of JA at each time point analyzed in comparison to the control (Figure 7D). Similarly, OPDA, H_2_JA, and JA-Ile levels increased along with aphid infestation (Figure 7D). JA biosynthesis genes (Figure 7E) up-regulation was also observed in *SbPRE4* OE lines, thus confirming the role of this gene in regulating JA biosynthesis. Subsequently, we treated the sorghum aphid-susceptible line 7B and Col-0 with 0.1 μM exogenous JA during aphid infestation; there was a significant decrease in the aphid number compared with the control (Figure 8A,B). These results suggest that *SbPRE4* enhanced plant aphid resistance via enhancing JA concentration.

## 3. Discussion

*Paclobutrazol-Resistance* (*PRE*) genes represent a class of genes encoding proteins that counteract the growth regulator paclobutrazol by inhibiting GA synthesis. *PRE* belongs to an atypical bHLH subfamily [11]. Despite the crucial roles *PREs* play in plant hormonal signaling and stress response, their presence and functional characteristics in sorghum remain unknown. This research conducted a comprehensive investigation and functional characterization of the *SbPRE* gene family utilizing bioinformatics tools and plant genetic modification biotechnologies. By integrating these findings with established functional studies in other species, this study offers insights for future investigations into sorghum *PRE* genes and paves the way for the identification of *SbPRE* genes associated with key resistant traits.

Research on the *PRE* genes has been conducted in various major crops such as *Oryza sativa*, *Gossypium hirsutum*, *Helianthus annuus*, and *Triticum aestivum* [25]. In sorghum, seven *PRE* genes were identified, indicating the conservation of this gene family across plant species. The gene structure and functional domains of SbPREs were elucidated through sequence analysis (Figure 1 and Figure 2B). It is known that the bHLH family is divided into DNA-binding bHLH (typical bHLH) and non-DNA-binding bHLH (atypical HLH). The SbPRE proteins belong to atypical bHLH transcription factors, which lack the DNA binding domain (Table 1 and Figure 1); therefore, they do not function as transcription factors. However, they can negatively regulate other bHLH transcription factors by forming heterodimers with them through the C-terminal HLH region, regulating gene expression.

RNA-seq data revealed that a significant role of *SbPREs* during growth and developmental processes, including *SbPRE2*, *SbPRE3*, *SbPRE4*, and *SbPRE7* exhibited high expression levels in root, shoot, leaf, inflorescence, and seed organs (Figure 4). The findings aligned with previous investigations across diverse plant tissues. In *Arabidopsis thaliana*, *AtPRE1* altered germination, the elongation of the hypocotyl/petiole, floral induction, and fruit development by regulating gibberellin (GA) [26]. In *Gossypium hirsutum*, *PRE* genes such as GhA09G0192 (*GhPRE1*), GhD09G0182, GhA07G1964, and GhD07G2183 exhibited abundant expression in floral tissues and *GhPRE1* was a positive regulator of fiber elongation [25]. In rice, the *PRE* gene *OsILI6* was mainly expressed in the root, and could also be detected in the pistil, lemma, palea, and young panicle, while it was almost absent in leaves and pistils [27]. Besides cereal crops, *PRE* genes play similar roles in horticulture plants. In strawberries (*Fragaria ananassa*), *FaPRE1* was predominantly expressed in ripe receptacles rather than in vegetative tissues [28]. In tomatoes (*Solanum lycopersicum* Mill. cv. *Ailsa Craig*), the *PRE* genes expression profiles were different. For example, *SlPRE1* was specific to flowers, the peak of *SlPRE2* expression was at the 10th day after aphid inoculation, *SlPRE3* showed low abundance, *SlPRE4* was highly expressed in hypocotyl and vegetative tissues, and *SlPRE5* displayed expression across multiple tissues [29]. These varying tissue-specific expression patterns of *PREs* in different plant species imply their involvement in diverse biological processes, suggestive of intricate and multifaceted functions.

Previous studies showed that *PREs* are involved in hormonal signaling. *AtPRE2* and *AtPRE6* in Arabidopsis play a significant role in ABA-mediated salt response. The expression levels of six *AtPRE* genes were decreased upon ABA treatment but elevated when exposed to salt stress [17]. In addition, *AtPRE6* was negatively modulated in the Auxin signaling pathway, while it was positively regulated in the ABA and salt signaling pathways [30]. In strawberries (*Fragaria ananassa*), *FaPRE1* could be repressed by IAA and activated by ABA, but the expression was almost unaffected by GA [28]. Moreover, *MdPRE4.3* owned the potential to enhance apple response to NaCl, ABA, and IAA, as well as enhance BR tolerance, while it almost does not affect the GA response [19]. In this study, the analysis of the *SbPRE* promoter indicated the presence of diverse *cis*-elements, including phytohormones and abiotic stress responsive elements (Figure 5). Additionally, various abiotic stresses (e.g., NaCl, drought, and heat) could influence *SbPRE* expression, suggesting its involvement in abiotic stresses in sorghum (Figure 6). However, the regulation mechanism of *PREs* in biotic stress remains unclear. We cloned *SbPRE4* and overexpressed it in transgenic Arabidopsis for further experiments. The results showed that *SbPRE4* enhanced Col-0 aphid resistance by accumulating JAs (Figure 7). As a reconfirmation, exogenous JA reduced aphid numbers on Arabidopsis and sorghum plants after aphid infestation for 7 days (Figure 8), suggesting that *SbPRE4* may be a positive regulator of JA regulating aphid resistance.

In summary, this study comprehensively investigated the *PRE* family genes in sorghum. The expression of the *SbPRE* genes in various sorghum tissues and under diverse stress conditions was thoroughly examined using RNA-seq data; moreover, the function of the *SbPRE4* gene, which was related to aphid resistance, was analyzed and confirmed through transgenic Arabidopsis. These findings substantially advance our comprehension of the *SbPRE* genes and will provide better resources for the future study and exploration of the *PRE* family genes in sorghum and other plant species.

## 4. Materials and Methods

### 4.1. Identification of the PRE Genes in Sorghum

In this study, the updated version of the *Sorghum bicolor* v3.1.1 genome was obtained from phytozome v13 (https://phytozome-next.jgi.doe.gov/ (accessed on 21 May 2024)) [31]. Blastp was used to identify all members of the *SbPRE* gene family. Protein homology alignment was conducted using *Arabidopsis thaliana* PRE protein sequences downloaded from the TAIR database (https://www.arabidopsis.org/ (accessed on 21 May 2024)) as the query sequence [32], including AtPRE1 (At5g39860), AtPRE2 (At5g15160), AtPRE3 (At1g74500), AtPRE4 (At3g47710), AtPRE5 (At3g28857), and AtPRE6 (At1g26945). The identified sorghum sequences were validated for conserved domains via submission to SMART (http://smart.embl-heidelberg.de/ (accessed on 21 May 2024)) [33], confirming them as candidate SbPREs. The gene length of *SbPREs* was extracted from the GFF3 annotation file of the reference genome. Additionally, predictions of the SbPRE protein sequences length, molecular weight, and isoelectric point (pI) were generated by ExPasy (https://www.expasy.org/ (accessed on 21 May 2024)). Furthermore, the bHLH structural domain localization within SbPREs was determined utilizing the Pfam database.

### 4.2. SbPRE Chromosomal Localizations and Gene Structures

To extract and visualize the chromosomal locations of the putative SbPREs, we utilized the Gtf/Gff3 sequence extraction tool and Gene Location Visualization function provided by TBtools [34]. By cross-referencing the sorghum gff3 file annotation, we were able to identify and visually represent the UTR regions, exon positions, and intron positions of the SbPRE genes using TBtools.

### 4.3. Structural Domains, Phylogenetic Analysis, and Promoters Analysis of SbPREs 

The Arabidopsis thaliana PRE sequences were sourced from the investigation conducted by Petroni et al. [35]. According to the existing literature, a collective 24 *PRE* genes were identified, seven for sorghum (*S. bicolor*), three for rice (*O. sativa*), eight for cotton (*G. hirsutum*), and six for Arabidopsis (*A. thaliana*). Sequence alignment was carried out using MUSCLE, which is available at www.ebi.ac.uk/Tools/msa/muscle/ (accessed on 21 May 2024). The phylogenetic tree was constructed utilizing the maximum likelihood approach in the MEGA 7.0 software, and its robustness was evaluated through bootstrap analysis comprising 1000 iterations. The resulting tree was visualized by the online tool Evolview v3 [36]. The entirety of the sorghum genome sequence was acquired from the Phytozome v13 database. The 2000 bp sequences preceding the transcription initiation site of the seven *SbPRE* genes were isolated as potential promoter sequences. The promoters were subjected to analysis for cis-acting elements associated with stress responsiveness and plant hormones using the PlantCARE software (http://bioinformatics.psb.ugent.be/webtools/plantcare/html/ (accessed on 21 May 2024)). The results were visualized by TBtools.

### 4.4. Expression Analysis of SbPREs by RNA-Seq Data or qRT-PCR

The raw transcriptome data of sorghum tissues were retrieved from the EBI database under the accession number ERP024458 [22]. Additionally, gene expression profiles in response to salinity, drought, heat, and aphid stresses were acquired from publicly available RNA-seq datasets [23]. The HISAT2 software (v2.2.0) was employed for aligning reads to the *S. bicolor* genome with default settings, and reads quantification was performed using StringTie software (v2.2.1), both of which were facilitated by TBtools. The normalization of gene expression levels was carried out utilizing the TPM (transcripts per kilobase million) method. The visualization of gene expression patterns was achieved through the utilization of the Super Heatmap browser tool within TBtools.

For qRT-PCR, the RNA was isolated as described [37]. AT1G32200 (*Actin1*) was used as the Arabidopsis internal gene. AT3G25760 (*AOC1*), AT5G42650 (*AOS*), AT1G72520 (*LOX4*), AT2G06050 (*OPR3*), JA biosynthesis genes, were detected. All primer sequences can be found in the research of Huang et al. [38].

### 4.5. Plant Materials and Aphid Treatments

The Columbia-0 (Col-0) wild-type Arabidopsis was employed for plant transformation. Both wild-type and transgenic Arabidopsis plants were germinated and cultivated in soil-filled pots. The growth conditions included a temperature of 22 °C, a photon flux density of approximately 125 μmol m^−2^ s^−1^, and photoperiods of 16 h light/8 h dark for long-day conditions and 8 h light/16 h dark for short-day conditions within a growth chamber. The aphids used for Arabidopsis were *M. persicae*; for detailed information refer to Moran and Thompson [39]. The 28-day-old Col-0 and OE *SbPRE4* transgenic Arabidopsis were inoculated with one wingless adult aphids. Plants were individually caged and aphids were counted 7 days later.

The sorghum aphid-susceptible line 7B was used. The seeds were planted in plastic containers filled with a soil mixture composed of vermiculite/organic substrate/loam (1:1:1 *v*/*v*/*v*). After germination, plants were grown in a greenhouse set at a constant temperature (28 ± 2 °C) with a 14/10 (light/dark) photoperiod and 60% relative humidity. Plants at the three-leaf stage were inoculated with aphids (*M. sacchari*); for detailed information refer to Pant et al. [40]. The experiments were carried out in Shijiazhuang (Hebei Province, China) in 2023.

### 4.6. Construction of the SbPRE4 Expression Vector and Arabidopsis Transformation

The construction of the *35S:SbPRE4* vector involved ligating the polymerase chain reaction (PCR) product into the BamHI restriction site of the pRok2 vector driven by the cauliflower mosaic virus 35S promoter. Subsequently, this vector was transformed into the *Agrobacterium tumefaciens* strain GV3101. The full-length fragments of *SbPRE4* were amplified from Btx623 sorghum using the polymerase chain reaction (PCR). The cloning primers used were SbPRE4-F 5′ATCGAGGTGCTGTAGCTTCG3′ and SbPRE4-R 5′TGTCGTCGTCGTCTTCAGTG3′; qRT-PCR primers were SbPRE4-F1 5′ACGAGCTCATCTCCAAGCTG3′ and SbPRE4-R1 GCAGTTCCCAGATCAGTGCC.

The *35S:SbPRE4* vector was transformed into *Arabidopsis thaliana* (Col-0) using the floral dip method as described by Clough and Bent [41]. Transgenic plants were identified by their resistance to 25 mg/L kanamycin on MS medium and allowed to grow to maturity.

### 4.7. Quantification of Phytohormones and JA Treatment

Following aphid treatment, plant samples of Col-0 and OE SbPRE4 (approximately 0.1 g each) were immediately frozen in liquid nitrogen and subsequently ground into powder. These ground samples were then subjected to extraction using a mixture of methanol, water, and formic acid (15:4:1, *v*:*v*:*v*). The resulting extracts were filtered through a 0.22 μm PTFE filter (Anpel, Shanghai, China) and subsequently analyzed using an LC-ESI-MS/MS system. This system consisted of an HPLC Shim-pack UFLC SHI-MADZU CBM30A (Shimadzu, Shanghai, China) system in conjunction with an Applied Biosystems 6500 Triple Quadrupole MS instrument (ThermoFisher, Shanghai, China). The AB6500 QTRAP LC/MS/MS system, equipped with an ESI turbo ion-spray interface and controlled by Analyst 1.6 software by AB Sciex (Framingham, MA, USA), was operated in both positive and negative ion modes for analysis [42]. The analytical conditions were as follows: LC, column, Waters ACQUITY UPLC HSS T3 C18 (100 mm × 2.1 mm i.d., 1.8 µm); solvent system, water with 0.04% acetic acid (A) and acetonitrile with 0.04% acetic acid (B); gradient program, started at 5% B (0–1 min), increased to 95% B (1–8 min), then to 95% B (8–9 min), and finally returned to 5% B (9.1–12 min); flow rate, 0.35 mL/min; temperature, 40 °C; and injection volume: 2 μL. The ESI source operation parameters were as follows: ion source, ESI+/−; source temperature 550 °C; ion spray voltage (IS) 5500 V (Positive), −4500 V (Negative); and curtain gas (CUR) was set at 35 psi. The Metware phytohormone database (MetWare, Wuhan, China) was constructed based on a standard set of phytohormones.

For JA treatment, 28-day-old Col-0 and the sorghum susceptible line 7B at the three-leaf stage were sprayed with 0.1 μM JA along with aphid infestation. Arabidopsis and sorghum were treated for every two days and three days, respectively. Three biological replicates were conducted. 

### 4.8. Statistical Analysis

Each experiment was conducted with three biological replicates, and within each biological replicate, three technical replicates were performed. The data are presented as means ± SD. Different lowercase letters in the comparison between multiple groups indicate a significant difference at *p* < 0.05 based on a one-way analysis of variance (ANOVA). The mean grouping test used in the ANOVA was Tukey HSD. Student’s *t*-test: (* *p* < 0.05 and ** *p* < 0.01) was used to analyze the statistical significance of the two groups. Error bars represent the standard deviation.

## 5. Conclusions

In this study, seven *SbPRE* genes were identified in sorghum and were distributed on chromosome 1, 4, 5 and 6. Phylogenetic evolution, cis-acting elements, and expression analysis suggested that *SbPRE* genes might play crucial roles in sorghum adaption to environmental stresses. The expression level of *SbPRE4* was up-regulated by aphid treatment. Moreover, overexpressing *SbPRE4* enhanced Arabidopsis aphid resistance by improving JAs. Furthermore, exogenous JA can decrease the aphid population in Arabidopsis and sorghum significantly. These findings implied that *SbPREs*, especially *SbPRE4,* play pivotal roles in the response to aphid stress.

## Figures and Tables

**Table 1:** The information on PREs in sorghum.

Gene	Gene ID	Chromosome Location	CDS Length (bp)	Protein Length (aa)	bHLH Domain	Molecular Weight (Da)	pI
*SbPRE1*	Sobic.006G236600	Chr06:57805968–57807417	315	105	2–104	11,180.38	8
*SbPRE2*	Sobic.006G131900	Chr06:49616617–49621803	483	161	39–109	17,385.78	9.48
*SbPRE3*	Sobic.004G267400	Chr04:61174563–61177405	1080	360	153–237	37,275.27	6.35
*SbPRE4*	Sobic.001G488600	Chr01:75864770–75871892	279	93	1–92	10,304.59	6.57
*SbPRE5*	Sobic.001G488400	Chr01:75826747–75828379	261	87	1–86	9690.97	9.03
*SbPRE6*	Sobic.001G254100	Chr01:28215024–28215913	288	96	1–95	10,442.79	7.98
*SbPRE7*	Sobic.005G178400	Chr05:66069136–66070056	405	135	4–114	13,862.51	6.73

CDS: coding sequence; bHLH: basic helix–loop–helix; pI: isoelectric point; aa: amino acid; Da: dalton.

## Data Availability

All data, tables, and figures in this manuscript are original, and are contained within the article.
